# Persistent detection of SARS-CoV-2 RNA in patients and healthcare workers with COVID-19

**DOI:** 10.1016/j.jcv.2020.104477

**Published:** 2020-08

**Authors:** Saurabh Gombar, Marcello Chang, Catherine A. Hogan, James Zehnder, Scott Boyd, Benjamin A. Pinsky, Nigam H. Shah

**Affiliations:** a Department of Pathology, School of Medicine, Stanford, CA, United States; b Department of Medicine (Biomedical Informatics), School of Medicine, Stanford, CA, United States; c Department of Medicine (Hematology), School of Medicine, Stanford, CA, United States

**Keywords:** SARS−COV-2, COVID-19, RT-PCR, Healthcare workers

## Abstract

**Background:**

Current guidelines for returning health care workers (HCW) to service after a positive SARS-CoV-2 RT-PCR test and ceasing of transmission precautions for patients is based on two general strategies. A test-based strategy that requires negative respiratory RT-PCR tests obtained after the resolution of symptoms. Alternatively, due to the limited availability of testing, many sites employ a symptom-based strategy that recommends excluding HCW from the workforce and keeping patients on contact precautions until a fixed period of time has elapsed from symptom recovery. The underlying assumption of the symptom-based strategy is that waiting for a fixed period of time is a surrogate for negative RT-PCR testing, which itself is a surrogate for the absence of shedding infectious virus.

**Objectives:**

To better understand the appropriate length of symptom based return to work and contact precaution strategies.

**Study Design:**

We performed an observational analysis of 150 patients and HCW that transitioned from RT-PCR SARS-CoV-2 positive to negative over the course of 2 months at a US academic medical center.

**Results:**

We found that the average time to transition from RT-PCR positive to negative was 24 days after symptom onset and 10 % remained positive even 33 days after symptom onset. No difference was seen in HCW and patients.

**Conclusions:**

These findings suggest until definitive evidence of the length of infective viral shedding is obtained that the fixed length of time before returning to work or ceasing contract precautions be revised to over one-month.

## 1 Background

Health care workers (HCW) who test positive for SARS-CoV-2 via reverse transcriptase PCR (RT-PCR) of nasopharyngeal swab (NP) specimens are asked to self-quarantine and only return to work after symptoms resolve and/or a fixed duration of time has passed. Similarly, hospitalized patients with COVID-19 are subject to transmission-based precautions to limit nosocomial spread. The CDC provides guidance for HCW to return to work, and for discontinuation of transmission-based precautions in hospitalized patients, for confirmed or suspected COVID-19 infection [1]. Both of these guidelines, last updated on April 30th, offer two strategies to proceed with routine processes. A test-based strategy requires two consecutive negative respiratory RT-PCR tests obtained after the resolution of symptoms. Alternatively, due to the limited availability of testing, a symptom-based strategy recommends excluding HCW from the workforce and keeping patients on contact precautions until at least 3 days after symptomatic recovery, and at least 7 days since initial symptom onset.

The underlying assumption of the symptom-based strategy is that waiting for a certain period of time is a surrogate for negative RT-PCR testing, which itself is a surrogate for the absence of shedding infectious virus. There have been several case reports and small series exploring RT-PCR positivity in COVID-19 patients suggesting that individuals can remain SARS-CoV-2 RNA positive significantly after symptom resolution [[2], [3], [4]]. Quantitative data are lacking on the extent to which such “duration of time” based exclusion is adequate to reduce transmission risk. Therefore, It remains uncertain whether current guidelines for the duration of exclusion from work and duration of contact precautions are adequate to reduce transmission.

## 2 Objectives

To better understand if the guidance to return HCW to routine activities in the absence of negative RT-PCR testing is appropriately timed by analyzing how long HCW remain positive for SARS-CoV-2.

## 3 Study design

We performed an observational analysis of 150 patients and HCW that transitioned from RT-PCR SARS-CoV-2 positive to negative over the course of 2 months at a large US academic medical center.

## 4 Results

We analyzed 33,038 RT-PCR tests performed at Stanford Healthcare (SHC) between 3/4/2020 and 4/30/2020. Tests were initially available to patients who experienced COVID related symptoms (cough, fever, runny nose, etc), travel to or through a region with known endemic COVID spread, close contact with confirmed SARS-CoV-2 infection, or HCW with suspected COVID exposure. After 3/17 testing was opened up to all individuals seeking care given physician discretion to order the lab test. Testing was performed using one of three emergency use authorized RT-PCR tests deployed at SHC [5,6]. Over this period 1809 (5.4 %) positive tests were obtained. Of this group, 87 patients and 63 healthcare workers had an initial positive test, follow up testing, and eventual negative test. Individuals were not included in the study if the time to their first follow-up test was greater than 1 standard deviation from the average time of a follow-up test in our dataset (cutoff of 20 days). The 150 individuals included had 380 NP RT-PCRs (2.5 tests on average per person) (Table 1).

**Table 1:** Selected demographics of patients and healthcare workers.

Category	Patients	HCW
Count	87	63
Age (SD)	57.2 (21.7)	35.5 (11.1)
<65	58	60
>=65	29	3
Female (%)	48.7	67.2
Mean Onset of Symptoms prior to the first test	7.9 (5.3)	4.6 (4.8)
Average time to first follow up	17.1	18.5
Median time to first negative test	25	23

For the patients, the average age was 57.2 years (standard deviation 21.7), with 48.7 % female. The average time from the first positive test to follow up testing was 17.1 days, and the median time for a patient to transition to negative was 25 days. For the HCW the average age was 35.5 years (standard deviation 11.1), with 67.2 % female. The average time from the first positive test to follow up testing was 18.5 and the median time for a patient to transition to negative was 23 days.

**Fig. 1:**
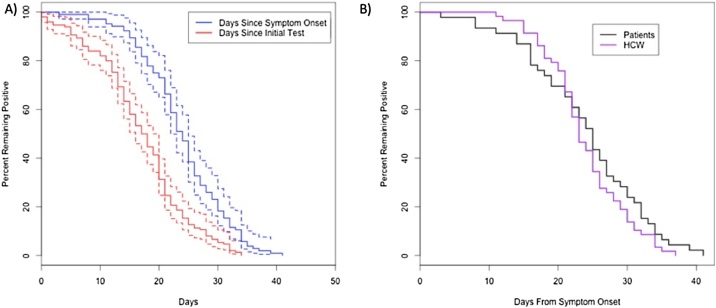
Duration of time for a positive RT-PCR test to turn negative. A) 150 individuals had an initial positive RT-PCR NP test followed by an eventual negative RT-PCR NP test. The median and 90th percentile for the duration of time between the positive to negative transition was 17.5 and 28 days respectively. From the time of symptom onset, the median and 90th percentiles were 24 days and 33 days respectively. Dashed lines represent the 95 % confidence interval. B) No difference was noted between 87 patients and 63 HCW in terms of duration of time for which the RT-PCR test remained positive after symptom onset.

The median time to transition from a positive test to a negative test was 17.5 days and 90 % of individuals had a negative RT-PCR test within 28 days of their first positive (Fig. 1a). Day of symptom onset was available for 80.1 % (121/150) of individuals, and the mean onset of symptoms was 6.1 days prior to the first positive test. From onset of symptoms, the median time to a negative RT-PCR was 24 days and the 90th percentile was 33 days. We found no demonstrable difference in the duration for which RT-PCR remained positive for HCW and patients (Fig. 1b).

## 5 Discussion

To date guidance for exclusion from work and length of contract precautions for patients with SARS-CoV-2 have been based on extrapolation of data from other infectious diseases. In recent weeks several publications from patient cohorts in China have demonstrated a similar length of RT-PCR positivity in both prospective and observational studies [7,8]. Our analysis is the first large cohort analysis of length of RT-PCR positivity in patients and HCW within the United States. Our findings suggest that a high proportion of individuals are persistently positive for SARS-CoV-2 RNA in NP specimens. Taking into account the mean duration of self-reported symptoms, 20 % of individuals remain RT-PCR positive for more than one month from symptom onset, and 10 % of the patients did not have a negative test until after 33 days had passed.

Our study, and similarly all studies based on RT-PCR analysis do not directly measure infectivity. Our analysis could be overestimating the length of infectious spreading by detecting non-infectious viral shedding. Large trials that rely on methods that detect the infective virus (ie viral culture) have not yet been reported in the literature. Until such studies are completed we suggest that return to work and contact precaution guidelines should require negative PCR tests or assume viral shedding for 33 days following symptom onset.

## Declaration of competing interest

None.
